# Patterns and Drivers of Vertical Distribution of the Ciliate Community from the Surface to the Abyssopelagic Zone in the Western Pacific Ocean

**DOI:** 10.3389/fmicb.2017.02559

**Published:** 2017-12-19

**Authors:** Feng Zhao, Sabine Filker, Kuidong Xu, Pingping Huang, Shan Zheng

**Affiliations:** ^1^Department of Marine Organism Taxonomy and Phylogeny, Institute of Oceanology, Chinese Academy of Sciences, Qingdao, China; ^2^Department of Molecular Ecology, University of Kaiserslautern, Kaiserslautern, Germany; ^3^University of Chinese Academy of Sciences, Beijing, China; ^4^Jiaozhou Bay Marine Ecosystem Research Station, Institute of Oceanology, Chinese Academy of Sciences, Qingdao, China

**Keywords:** ciliate community, protists, high throughput sequencing, the Western Pacific Ocean, abyssopelagic zone, stratification

## Abstract

The deep sea is one of the largest but least understood ecosystems on earth. Knowledge about the diversity and distribution patterns as well as drivers of microbial eukaryote (including ciliates) along the water column, particularly below the photic zone, is scarce. In this study, we investigated the diversity of pelagic ciliates, the main group of marine microeukaryotes, their vertical distribution from the surface to the abyssopelagic zone, as well as their horizontal distribution over a distance of 1,300 km in the Western Pacific Ocean, using high-throughput DNA and cDNA (complementary DNA) sequencing. No distance-decay relationship could be detected along the horizontal scale; instead, a distinct vertical distribution within the ciliate communities was revealed. The alpha diversity of the ciliate communities in the deep chlorophyll maximum (DCM) and the 200 m layer turned out to be significantly higher compared with the other water layers. The ciliate communities in the 200 m water layer appeared to be more similar to those in deeper layers from 1,000 m to about 5,000 m than to the surface and DCM ciliate communities. Dominant species in the bathypelagic and abyssopelagic zone, particularly some parasites, were also detected in the 200 m layer, but were almost absent in the surface layer. The 200 m layer, therefore, seems to be an important “species bank” for deep ocean layers. Statistical analyses further revealed significant effects of temperature and chlorophyll a on the partitioning of ciliate diversity, indicating that environmental factors are a stronger force in shaping marine pelagic ciliate communities than the geographic distance.

## Introduction

Microbial eukaryotes, which cover a wide spectrum of cell shapes and taxonomic affiliations, dominate the domain Eukaryota in terms of diversity and abundance (e.g., Caron et al., 2012; Massana et al., 2015). They play a variety of critical roles in marine systems as primary producers, predators, decomposers, and parasites (Sherr and Sherr, 2002, 2009; Fenchel, 2008). However, the estimation of diversity and geographic distribution of microbial eukaryotes has been a topic of interest in the scientific community for a long time. Finlay (2002) strongly indicated that microbial eukaryotes are so abundant that continuous large-scale dispersal sustains their global distribution, and awarded them a cosmopolitan status. In contrast, Foissner (1999, 2008) postulated a moderate endemicity model, and proposed that the vast majority (>90%) of microbial eukaryotes exhibit moderate or low abundance, and therefore, are biogeographically restricted (endemic species).

Ciliates, with their high diversity and wide distribution, as well as a broad range of body sizes, are the taxa that triggered the debate of microbial diversity and distribution. However, several of the important arguments in the debate have been mainly derived from the observations of freshwater and soil ciliates. Marine ciliates have long been considered cosmopolitans, whose distributions are also driven by ocean currents and circulation. In marine ecosystems, pelagic ciliates are important components; they episodically dominate the microzooplankton and play a pivotal role in the multi-step microbial food web (Pierce and Turner, 1992; Agatha, 2011). Due to their functional importance as well as the scientific debate related to ciliate diversity and distribution, an increasing amount of research has focused on the investigation of community patterns of pelagic ciliates. Most studies have focused on pelagic ciliates in the photic zone of coastal regions (Doherty et al., 2010; Santoferrara et al., 2011; Grattepanche et al., 2014), and continental shelves (Santoferrara and Alder, 2012; Yu et al., 2014). A series of studies have also been conducted in the open sea area of the Pacific Ocean (Dolan et al., 2007; Gómez, 2007), the Atlantic Ocean (Montagnes et al., 2010), the Antarctic Ocean (Dolan et al., 2012), and the Mediterranean Sea (Pitta et al., 2001; Christaki et al., 2011). In particular, the recent Tara Oceans expedition has shed light on our understanding of the global diversity and distribution patterns of pelagic ciliates within the photic zone (de Vargas et al., 2015; Gimmler et al., 2016). These studies revealed an increase in ciliate abundance and species richness down to the chlorophyll maximum zone, as well as a wide geographic distribution of a high proportion of pelagic ciliates (e.g., Dolan et al., 2012; Gimmler et al., 2016).

In contrast, research on the patterns of microeukaryotic diversity throughout the water column, particularly below the mesopelagic zone (down 1,000 m), is just in its nascent phase (Pernice et al., 2015, 2016). The few available studies on pelagic ciliates below the photic zone and to a depth of 800 m uncovered different trends and distribution patterns. Wickham et al. (2011) revealed a decrease in ciliate abundance and species richness with increasing depth below the DCM in the Antarctic Amundsen and Bellingshausen Seas. Likewise, less ciliate OTUs were detected below the photic zone than above it (Countway et al., 2010). In contrast, Grattepanche et al. (2016) detected an unexpected increase in ciliate diversity with increasing depth up to 800 m off the coast of New England, United States. Hu et al. (2016) found a similar pattern at a coastal ocean station in the eastern North Pacific, and indicated that ciliates displayed previously unreported high relative activities below the photic zone. Overall, the true patterns of pelagic ciliate diversity in the deep layers and the changes in community composition along a depth gradient are still far from being fully understood.

High-throughput DNA sequencing has contributed substantially to the detection of the wide-ranging protist diversity. However, the microbial diversity detected by DNA sequencing likely includes not only active organisms, but also extracellular DNA, dead cells, and resting stages of organisms (Stoeck et al., 2007). This needs to be considered while working in marine environments, especially when working with sediments or deeper water layers, as these zones could become sinkholes for free DNA and dead organisms originating from the higher water layers. In contrast, due to the high instability of the RNA-structure, the decay of these molecules is quite rapid compared with DNA (Stoeck et al., 2007). Therefore, RNA-based molecular surveys identify theoretically active protist communities, and might result in a different picture of biodiversity compared with the DNA surveys (Massana et al., 2015). Recent studies have started to examine the differences in marine protist community structures derived from DNA or RNA samples. While no significant difference was detected in the community structure of protists in the coastal waters and sediments (Massana et al., 2015), obvious variations were found in the sediments from deep-sea hydrothermal vents and adjacent sea areas between DNA and cDNA (complementary DNA, synthesized from an RNA template) sequence analysis (Zhao and Xu, 2016). The available data indicate that different habitats might lead to alterations in the levels of similarity between the community structures derived from DNA and cDNA sequencing. Empirical results regarding the effect of DNA or cDNA-based techniques on the community composition of pelagic microeukaryotes in the bathypelagic and abyssopelagic zones are, thus far, not available.

Using high-throughput sequencing, our study aimed to (i) assess the suitability of DNA versus RNA as genetic signature to decipher marine ciliate community structures, (ii) investigate the vertical distribution pattern of marine ciliates from the surface to the abyssopelagic zone until a depth of 5,000 m, and their horizontal distribution pattern across a distance scale of 1,300 km in the Western Pacific Ocean, and (iii) identify the environmental factor(s) responsible for shaping the ciliate communities in the open ocean.

## Materials and Methods

### Sample Collection and Environmental Measurements

**FIGURE 1:**
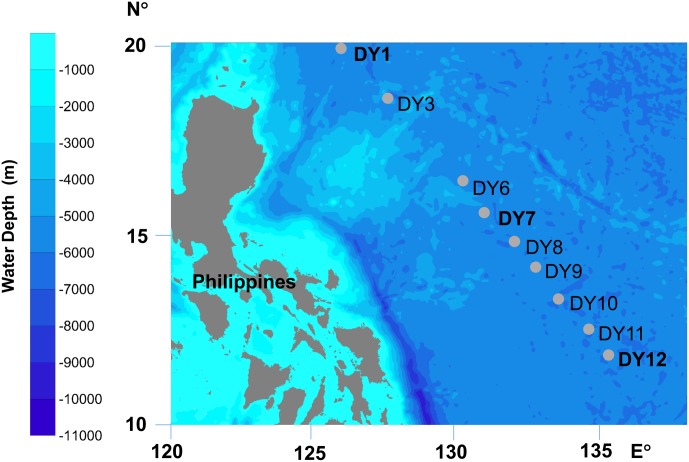
Locations of sampling stations. At stations DY1, DY7, and DY12, water samples were collected at five depths: surface (Sur), deep chlorophyll maximum (DCM), 200, 1,000, and 2,000 m, and the seawater were also sampled at the bottom layer of 4981 m at DY7 and 4765 m at DY12. At the other six stations of DY3, 6, 8, 9, 10, and 11, only the surface seawater was collected.

Seawater samples were collected from nine stations situated in the Western Pacific Ocean in December 2015 (**Figure 1** and **Table 1**). Horizontal surface water samples were taken from a depth of 2 m at all nine stations along a distance of 1,300 km. Vertical depth gradients were sampled at three of the nine stations (DY1, DY7, and DY12) at the following depths: surface, deep chlorophyll maximum (DCM), 200, 1,000, and 2,000 m. Additionally, at stations DY7 and DY12, bottom seawater samples were also taken at a depth of 4,981 and 4,765 m, respectively (**Table 1**).

**Table 1:** Location and environmental parameters of water samples collected from the Western Pacific Ocean.

Samples	Longitude	Latitude	Maximum water depth (m)	Sampling depth (m)	Salinity	Temperature (°C)	Chlorophyll a (μg/L)
DY3.Sur	127°42′ E	18°36.6′ N	5023	2	34.7	27.8	0.105
DY6.Sur	130°15.6′ E	16°24′ N	6589	2	34.5	28.0	0.086
DY8.Sur	132°8.4′ E	14°51.6′ N	5609	2	34.2	28.0	0.070
DY9.Sur	132°49.8’ E	14°9′ N	5840	2	34.5	28.5	0.081
DY10.Sur	133°42.6’ E	13°18.6′ N	5153	2	34.1	28.8	0.133
DY11.Sur	134°42.4′ E	12°31.2′ N	3826	2	34.2	28.7	0.132

DY1.Sur	126°0.6′ E	20°9.6′ N	6158	2	34.6	27.6	0.114
DY1.DCM				106	34.6	27.6	0.280
DY1.200				200	34.9	20.7	0.023
DY1.1000				1000	34.5	4.20	0.001
DY1.2000				2000	34.6	2.10	0.001

DY7.Sur	131°7.8′ E	15°35.4′ N	5231	2	34.5	28.3	0.055
DY7.DCM				90	34.9	26.4	0.322
DY7.200				200	34.7	17.3	0.006
DY7.1000				1000	34.5	3.89	0.002
DY7.2000				2000	34.6	1.98	0.001
DY7.B				4981	34.7	1.67	0.001

DY12.Sur	135°21′ E	11°52.2′ N	4860	2	34.5	28.6	0.052
DY12.DCM				96	35.2	23.8	0.199
DY12.200				200	34.5	13.5	0.024
DY12.1000				1000	34.5	4.64	0.002
DY12.2000				2000	34.6	2.30	0.001
DY12.B				4765	34.7	1.58	0.001


Seawater was collected with Niskin bottles attached to a rosette sampling system equipped with a Seabird CTD probe. At each layer of each station, 20 L of seawater was pre-filtered through a 200-μm sieve and then filtered through polycarbonate filters (0.22-μm pore size) to collect the environmental DNA and RNA. The filters were immediately shock frozen in liquid nitrogen and then stored at -80°C until further processing in the lab. A total volume of 1.8 L water was collected at each station and each layer for measuring the concentration of Chlorophyll a. Chlorophyll a was extracted in 90% acetone at 4°C for 24 h, and measured using a Turner Designs Trilogy (Turner Designs, United States) (Parsons et al., 1984). Total nitrogen (TN) and total phosphorus (TP) in seawater were determined after persulphate oxidation with a continuous flow analyzer (QuAAtro, Seal Analytical Limited, United Kingdom), according to the manufacture’s manual. The concentrations of NO_3_-N, NO_2_-N, PO_4_-P, SiO_3_-Si, and NH_4_-N in the GF/F filtrate were analyzed according to the Joint Global Ocean Flux Study (JGOFS) spectrophotometric method (Gordon et al., 1993), with a continuous flow analyzer (QuAAtro, Seal Analytical Limited, United Kingdom).

### DNA and RNA Extraction, Reverse Transcription

Environmental DNA was extracted from all samples using Qiagen’s All Prep DNA/RNA Mini Kit (Qiagen, Germany) according to the manufacturer’s instructions. Additionally, using the same kit, total RNA was extracted from all the samples of station DY12. Three subsamples of total RNA from each layer of DY12 were reverse-transcribed into cDNA using the 1st strand cDNA synthesis kit (TAKARA BIO INC., Japan) with the random primers provided in the kit.

### PCR Amplification

Three DNA and cDNA subsamples of each sample were used to amplify the hypervariable V4 region of the ciliate 18S rRNA gene in a nested PCR approach (Stock et al., 2013). First, amplification with ciliate-specific primers CilF and CilR I–III was performed, in order to specifically amplify the ciliate 18S rRNA gene (Lara et al., 2007). Subsequently, the purified PCR products from the first reaction were subjected to a second PCR, which adopted eukaryote-specific primers for the amplification of the hypervariable V4 region (Stoeck et al., 2010). To minimize the PCR-bias, three replicate PCRs were conducted for each sample and pooled afterward.

### High-Throughput Sequencing and Data Processing

Sequencing libraries were constructed using the NEB Next^®^ Ultra^TM^ DNA Library Prep Kit for Illumina (NEB, United States). The quality of the libraries was assessed with a Qubit 2.0 Fluorometer (Thermo Scientific, United States) and an Agilent Bioanalyzer 2100 system. Paired-end sequencing (2 × 300 bp) of the libraries was conducted on an Illumina MiSeq platform by Novogene Bioinformatics Technology Co., Ltd. The ciliate sequence reads have been deposited at the National Center for Biotechnology Information (NCBI) Sequence Read Archive under accession number SRP109014.

Paired-end reads were merged using FLASH v1.2.7 based on overlapping regions within corresponding reads (Magoč and Salzberg, 2011), and filtered according to the QIIME quality control process using the following settings: maximum number of consecutive low quality base = 3, minimum proportion of continuous high-quality base = 75% (Caporaso et al., 2010). After filtering, unique reads were identified and the number of occurrences for each read was recorded by UPARSE (Edgar, 2013). Reads occurring only once (singletons) were discarded. Using UPARSE, a sequence similarity of 97% was used to delineate ciliate OTUs (Nebel et al., 2011; Dunthorn et al., 2012; Massana et al., 2015; Forster et al., 2016). The taxonomic information for the representative sequence of each OTU was analyzed using BLAST against the SILVA database (v. 123), as implemented in QIIME v. 1.9.0 (Caporaso et al., 2010).

We defined OTUs as rare when they represented ≤ 0.1% of all the sequences in a sample, and abundant when they represented ≥ 1% of all sequences in a sample (Fuhrman, 2009; Pedrós-Alió, 2012). Moderately abundant OTUs were defined as those with a sequence proportion ranging between the rare and abundant OTUs.

### Statistical Analyses

Rarefaction curves were used in order to investigate the degree of sample saturation. To ensure inter-sample comparability for our taxonomic richness estimates and statistics, the number of sequences per sample was normalized to the smallest sample size (*n* = 9,513). Alpha diversity of the samples was assessed based on OTU richness, Shannon H’, and effective number of species [exp(H’)]. To predict the ciliate OTU richness, incidence-based (ICE) coverage estimator, which avoids biases induced by uneven gene copy numbers among different ciliate taxa, was calculated. Differences in environmental parameters between samples were analyzed by calculating the Euclidean distances between the samples after a log(x+1) transformation of the environmental parameters. Euclidean distance (environmental parameters) values were then used for the unweighted pair-group method with arithmetic means (UPGMA) cluster analyses.

To determine the environmental factors driving the partitioning of ciliate diversity, Jaccard similarities were first transformed into a distance matrix for a non-metric multidimensional scaling (NMDS) analysis. Next, the environmental parameters were checked for redundancy and removed whenever the Spearman’s correlation revealed a significant ρ^2^ > 0.8 based on the analysis of varclus (R package “Hmisc,” Harrell, 2008). Thus, NO_3_-N, which was closely correlated with PO_4_-P, and depth, which was closely correlated with temperature, were removed. Non-redundant environmental parameters were then fitted to the NMDS ordination using the envfit function of the vegan package (Oksanen et al., 2010) in R with 10,000 permutations. The significance of the correlation between each non-redundant environmental parameter and the binary Jaccard similarity matrix were further tested by a permutational analysis of variance using the “adonis” function of the vegan package (Oksanen et al., 2010), with 10,000 permutations.

To display the differences in the community composition and structure within each layer, a heatmap analysis based on the 50 most abundant OTUs was performed using the ‘pheatmap’ package in R. Prior to this analysis, we calculated the average number of sequences in each layer of stations DY1, DY7, and DY12, as well as the relative proportion that contributed to any given layer for a given taxon. Moreover, the OTUs, which were responsible for the qualitative differences that caused the dissimilarity between the sample groups, were identified using the SIMPER function in PRIMER v6 (Plymouth Marine Laboratory, United Kingdom).

A potential distance-decay relationship was assessed by a Mantel test (Diniz-Filho et al., 2013) using the ‘vegan’ package in R (Oksanen et al., 2010). Therefore, pairwise community Bray-Curtis dissimilarities were calculated for the nine surface samples as well as for the deeper layers of stations DY1, DY7, and DY12, respectively, and were correlated with the geographic distance between pairs of stations.

The ANOSIM function in PRIMER v6 was used to test the differences detected by analyzing DNA and cDNA signatures. ANOSIM provides an R-statistic to evaluate the dissimilarity of groups. Thus, groups are dissimilar if the R-statistic is close to 1.

### Identification of Novel Diversity

To identify the novel ciliate diversity, we followed the workflow proposed by Filker et al. (2015). Briefly, the representative sequences of the detected OTUs were aligned using Seaview v.4.6.1 (Galtier et al., 1996) with alignment option “clusto”. Pairwise similarities of aligned sequences were then calculated with a custom script. Finally, the “igraph” package (Csárdi and Nepusz, 2006) was used to build networks based on the pairwise sequence similarities. The resulting networks were visualized and modified using GEPHI v.0.9.1 (Bastian et al., 2009).

## Results

### Environmental Parameters

The chlorophyll a concentrations in the surface layer ranged between 0.052 μg/L (DY12) and 0.133 μg/L (DY10; **Table 1**). The deep chlorophyll maxima (DCM) of stations DY7, DY12, and DY1 were detected with a concentration of 0.32 μg/L at 90 m, 0.199 μg/L at 100 m, and 0.28 μg/L at 106 m, respectively. From the surface to the bottom, the temperature decreased from 28.6°C (DY12) to 1.6°C (DY12), respectively. The concentrations of total phosphorus (TP), total nitrogen (TN), NO_3_-N, PO_4_-P, and SiO_3_-Si varied in the surface layer (**Supplementary Table S1**). The concentrations of these environmental parameters increased along the water column to the depth of 2,000 m (**Table 1** and **Supplementary Table S1**).

**FIGURE 2:**
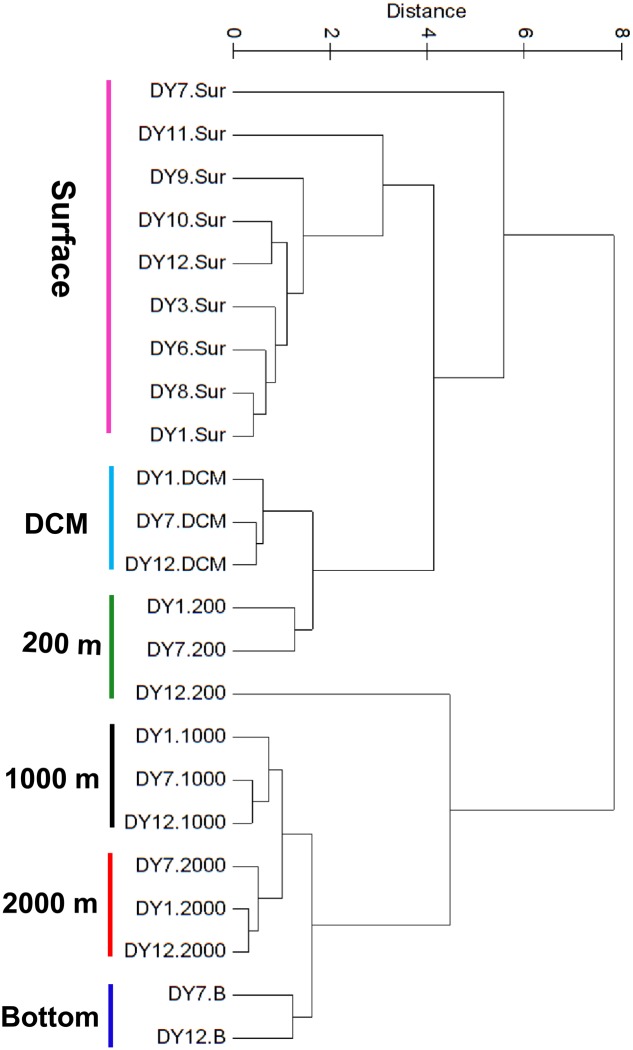
Unweighted pair-group method with arithmetic means (UPGMA) clustering of environmental parameters based on the Euclidean distance similarity matrices.

UPGMA cluster analysis of the environmental parameters revealed distinct groups, separating samples belonging to distinct water layers from each other (**Figure 2**). Only the surface sample of station DY7 and the 200 m sample of station DY12 could not be grouped in the analysis. Based on the environmental parameters, surface samples were more similar to the DCM and 200 m samples compared with all other samples. Samples from 1,000 and 2,000 m depth were more similar to each other than to the bottom water samples at 4981 and 4765 m depth.

### Overview of Sequencing Data

For the 23 DNA samples under study, a total of 739,909 high-quality ciliate V4 reads could be retrieved. Sequence numbers for each sample ranged between 9,513 (DY12.1000) and 64,187 (DY8.Sur), with an average of 32,169 sequences (**Supplementary Table S2**). Based on a clustering threshold of 97%, a total of 454 different ciliate OTUs were obtained. The total number of OTUs analyzed for each sample varied from 78 at station DY12.2000 to 219 at station DY12.DCM, with an average of 140 OTUs (**Supplementary Table S2**). Sequencing of the six cDNA samples of station DY12 yielded a total of 202,116 high-quality ciliate sequences (lowest sequence number: 22,823 for RDY12.DCM; highest sequence number: 42,885 for RDY12.B; average sequence number: 33,686), which clustered into 379 distinct OTUs (**Supplementary Table S2**). Rarefaction analyses of all DNA and cDNA samples indicated near-saturated sampling for all samples (**Supplementary Figure S1**).

### Alpha Diversity of Ciliate Communities

The ciliate OTU richness showed a rough decreasing trend from the northwest station DY1 to the southeast station DY12, whereas no trend was observed for either the Shannon index values or the effective number of species (**Supplementary Table S2**). Observed ciliate alpha diversity was stable across each water layer, but varied along the depth gradients (**Supplementary Table S2**). The effective number of species and OTU richness peaked in the DCM layer [mean exp(H’): 45 ± 12; mean OTU richness: 205 ± 11], followed by the 200 m depth layer [mean exp(H’): 35 ± 9; mean OTU richness: 186 ± 20] and surface water [mean exp(H’): 23 ± 6; mean OTU richness: 135 ± 24]. Alpha diversity was lowest in the 2,000 m depth samples [mean exp(H’): 14.97 ± 10; mean OTU richness: 107 ± 29]. Changes in the OTU richness were significant between the DCM and 200 m ciliate communities, respectively, and the communities of the other water layers (*p* = 0.0004–0.039). Samples of each station covered up to 45% (DY12.DCM) of the predicted overall OTU richness (ICE = 492 OTUs, slightly exceeding the total number of observed OTUs, with an average of 29% ± 8%). When the analysis was confined to the nine surface water samples, each sample covered 51% ± 9% of the predicted overall OTU richness (ICE = 267 OTUs); samples of the 2,000 m layer covered around 41% ± 11%, and samples of the DCM layer covered as much as 70% ± 4% of the predicted overall OTU richness for the respective layer.

### Abundant vs. Rare, Shared vs. Unique OTUs

**FIGURE 3:**
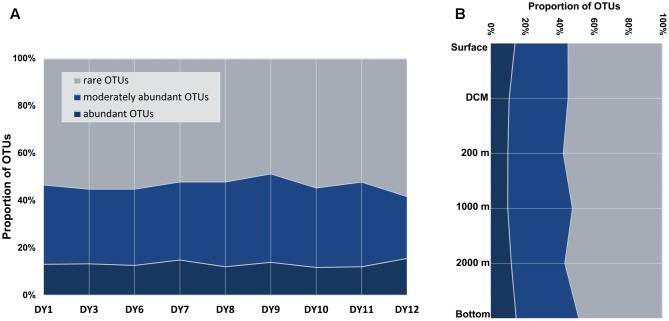
Proportions of abundant, moderately abundant and rare OTUs in all the surface samples **(A)**, and along the depths gradients **(B)** detected by DNA/cDNA sequencing.

Abundant ciliate OTUs accounted for 3% (DY1.2000) to 24% (DY12.2000), whereas rare OTUs contributed 31% (DY12.2000) to as much as 79% in sample DY1.2000 throughout all samples (**Figure 3**). On an average, the lowest proportion of abundant OTUs was observed in the samples from 200 and 1,000 m depth (mean value: 10% ± 2% and 10% ± 5%, respectively), whereas the highest proportion was observed in the bottom seawater samples (15% ± 4%). Likewise, the highest proportion of rare OTUs was detected in the 200 m samples (58% ± 2%) and the lowest was detected in the 1,000 m samples (52% ± 9%).

Thirteen of the identified OTUs were shared by all 23 DNA-based samples, and 103 OTUs (22%) were common in more than half of all samples. About 31% of the OTUs detected in our study were unique to one (17.2%) or two (13.9%) samples. When the analysis was confined to the nine surface water samples, 26.7% of the OTUs could be retrieved from all nine surface samples, whereas 24.3% were unique to one sample.

In terms of OTU distribution patterns at different layers, 12% occurred at all sampled layers (at least in one sample of each layer), and 28% occurred only at one layer. The proportion of unique OTUs at each layer was 5.2% at the surface layer, 14.4% in the DCM layer, 14.3% at the 200 m layer, 10.9% at the 1,000 m layer, and 7.1% at the 2,000 m layer (**Supplementary Figure S2**).

### Environmental Effects on Partitioning of Marine Pelagic Ciliate Diversity

**FIGURE 4:**
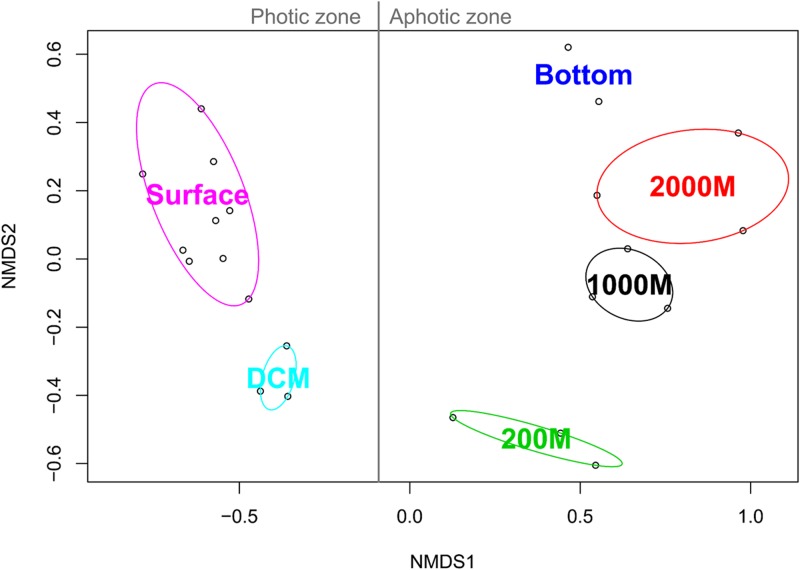
Non-metric multidimensional scaling analysis of the Western Pacific Ocean samples based on Jaccard similarities. For each community cluster, the ellipses represent the 95% confidence interval.

NMDS analysis based on the binary Jaccard index between the marine ciliate communities of each sample grouped all the samples according to their collection depth. All photic samples (surface, DCM) were separated from the aphotic samples (200, 1,000, 2,000 m, and deeper layers) by NMDS axis 1. Additionally, a second gradient was detected for the photic and aphotic samples, along NMDS axis 2 (**Figure 4**).

Fitting of the environmental parameters onto the NMDS revealed significant effects of all factors (*p* < 0.05) except NH_4_-N and salinity. Interestingly, most environmental factors showed a stronger affinity to NMDS axis 1, with NO_2_-N exhibiting the strongest correlation (**Table 2**). The highest significant affinity to NMDS axis 2 was found for SiO_3_-Si. After permutational analysis of variance, only temperature and chlorophyll a remained as explanatory variables for the partitioning of ciliate diversity (**Table 2**).

**Table 2:** Results of environmental factor fitting on ordination and permutational analyses of variance on distance matrices.

	Environmental factor fitting				Permutational analyses of variance	
		
Variable	NMDS1	NMDS2	*R*^2^	*P*	*R*^2^	*P*
Temperature	-0.92	-0.40	0.95	0.00001	0.20	0.001
Chlorophyll a	-0.71	-0.70	0.51	0.0003	0.06	0.023
Salinity	0.29	-0.96	0.17	0.15	0.08	0.006
Total phosphorus	0.95	0.32	0.67	0.0002	0.03	0.37
Total nitrogen	0.95	0.30	0.7	0.00001	0.04	0.18
PO_4_-P	0.70	0.71	0.8	0.0002	0.047	0.056
NO_2_-N	0.98	-0.19	0.47	0.002	0.049	0.07
SiO_3_-Si	0.69	0.73	0.79	0.00001	0.047	0.088
NH_4_-N	-0.01	1.00	0.24	0.061	0.038	0.225


### Effect of Geographic Distance on Surface Ciliate Community Structure

The Mantel test revealed no significant correlation between geographic distance and the pairwise community dissimilarities of total OTUs (*r* = 0.26, *p* = 0.13), abundant OTUs (*r* = 0.21, *p* = 0.16), moderately abundant OTUs (*r* = 0.17, *p* = 0.18), or rare OTUs (*r* = 0.12, *p* = 0.29) detected in the surface samples. No significant correlation was revealed between the pairwise community dissimilarity of the other layers and geographic distance either.

### Taxonomic Composition

**FIGURE 5:**
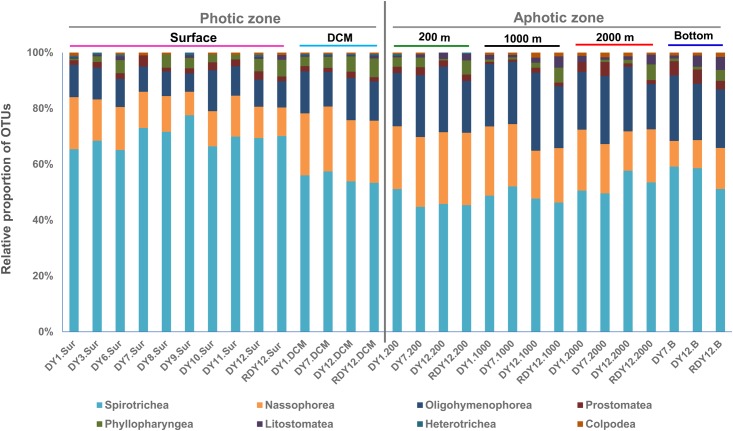
OTU-based taxonomic composition of ciliate communities in each sample.

The OTUs obtained from the 23 DNA-based samples were related to eight of the 11 ciliate classes: Spirotrichea, Oligohymenophorea, Nassophorea, Colpodea, Prostomatea, Litostomatea, Phyllopharyngea, and Heterotrichea (**Figure 5**).

Spirotrichea constituted the dominant ciliate group, contributing 46.8% overall to the OTUs, and up to 78% in a single community (DY9.Sur: 83 OTUs, **Figure 5**). Within Spirotrichea, subclasses Oligotrichia (30% of total OTUs in each sample on average) and Choreotrichia (25% of total OTUs in each sample on average) represented a high proportion of ciliate OTUs. The Spirotrichea sequences contributed an average 70.7% to the total sequences across all stations, and up to 97% at station DY9.Sur (**Supplementary Figure S3**). A higher proportion of the spirotrichean OTUs was found in the photic layers (66% on average), compared with the aphotic layers (55% on average).

Nassophorea were the second most diverse group, contributing 23.7% to the overall OTU diversity. In the photic layers, nassophorean OTUs accounted for 8–23% of the total OTUs in each sample, with an average of 16%. A slightly higher proportion (9–26%) was observed in the aphotic layers, with an average proportion of 19%. Nassophorean OTUs were the most diverse at the DCM and 200 m layers, while nassophorean sequences were the most abundant at the surface and DCM layers (**Figure 5** and **Supplementary Figure S3**).

Oligohymenophorea contributed to 17.8% to the overall OTU diversity. In photic layers, the proportion of the oligohymenophorean OTUs at different stations was 11% on average, ranging from 7 to 15%. Oligohymenophorea, in particular, some of its parasitic members, were more diverse and abundant in aphotic layers than in the photic layers, accounting for 19–28% of the total OTUs at each sample, with an average of 23%.

Prostomatea were also detected in all the 23 samples, and contributed to 2.3% of the overall OTU diversity (**Figure 5**). Prostomatean sequences generally contributed less than 1% to the total sequence number in each sample. Prostomatean OTUs were randomly distributed along the water column, and contributed an average 2 and 3% of the total OTUs in each sample from photic and aphotic layers, respectively.

Colpodea accounted for 0.6% (3 OTUs) of the overall OTU diversity (**Figure 5**), and were most abundant at stations DY1.2000 and DY12.2000, with a relative proportion of 41% and 48%, respectively. Litostomatea, Phyllopharyngea, and Heterotrichea generally accounted for less than 1% of the total OTUs each (**Figure 5** and **Supplementary Figure S3**).

The dissimilarities in ciliate communities between the photic group and the aphotic group were mainly due to changes in 22 OTUs, which contributed more than 1% to the total differences between the two groups of samples, as revealed by the SIMPER analyses. Among them, nine OTUs belonging to the subclass Oligotrichia were more abundant in the photic layers and 13 OTUs, including four OTUs closely related to parasitic ciliates, were more abundant in the aphotic layers.

**FIGURE 6:**
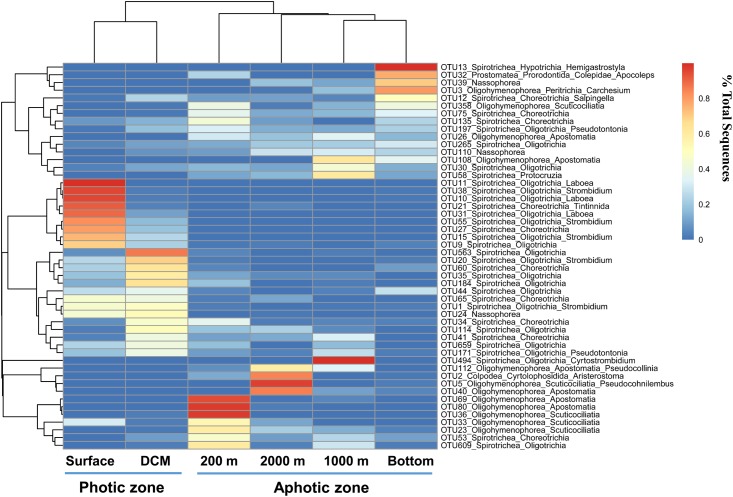
Heatmap clustering of different water layers (*x* axis) and the top abundant 50 OTUs (*y* axis). The color code represents the percentage contributed by a given OTU to any given water layers.

The heatmap of the most abundant 50 OTUs showed a similar pattern: most OTUs that were abundant in the photic layers belonged to Oligotrichia and Choreotrichia, whereas oligohymenophoreans were abundant in the aphotic layers (**Figure 6**). The heatmap also showed that most abundant OTUs at the photic layers (the surface and DCM) disappeared or accounted for only a minor proportion in the aphotic layers. By contrast, the 200 m layer and the other deep layers shared more OTUs at relatively high proportions (**Figure 6**).

### Degree of Novel Diversity

**FIGURE 7:**
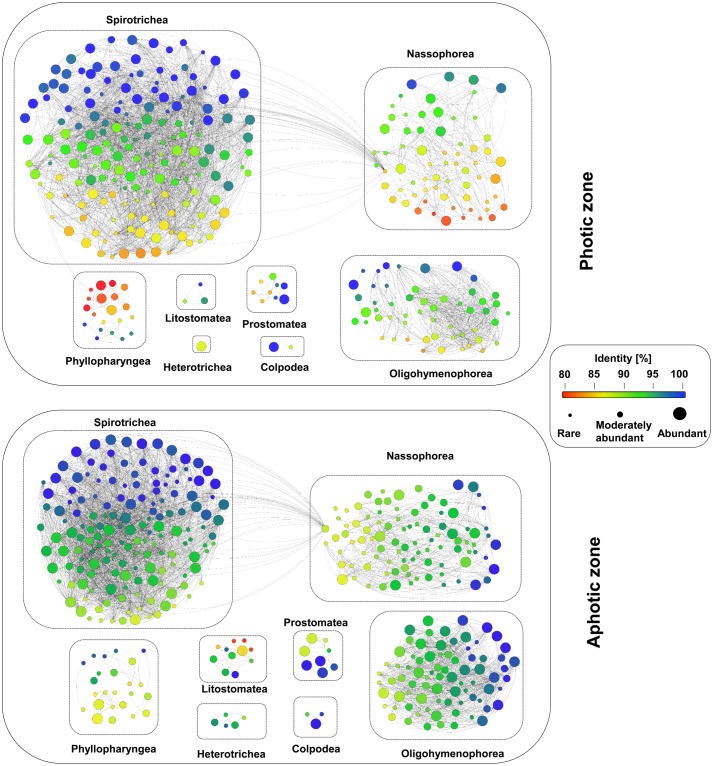
Analyses of the novel diversity within the ciliate communities in the waters of the Western Pacific Ocean. Each node represents one OTU, the different colors indicate the sequence similarity to a deposited reference sequence, and the size of the node indicates the levels of their relative abundance, namely abundant, moderately abundant and rare. An edge weight (sequence similarity) of 90% was chosen to discriminate between the different OTUs.

The observation of numerous low-identity ciliate OTUs in our dataset points to a high genetic novelty within the Western Pacific Ocean. The mean similarity of all detected ciliate OTUs to previously described sequences was only 95.24% (**Figure 7**). About 67% of the OTUs had a sequence similarity of less than 97% to the reference sequences and 42% of the OTUs had an identity match of less than 95% (**Figure 7**). The proportion of OTUs with similarities less than 97 or 95% was similar in each layer along the depth gradient, and none of the layers emerged as an extraordinary hotspot of uncharted ciliate diversity.

Abundant OTUs had a higher sequence similarity to reference sequences than moderately abundant and rare OTUs: 20% of the OTUs had the highest sequence similarity to a reference sequence (100%), whereas only 18% had a sequence similarity of less than 95% to reference sequences. However, some abundant OTUs, e.g., the most abundant nassophorean OTUs detected in photic layers, had a sequence similarity of less than 90% to the reference sequences (**Figure 7**). In contrast, as little as 5% of the moderately abundant OTUs and 3% of the rare OTUs revealed a sequence similarity of 100%. About 46% of the moderately abundant OTUs and 54% of the rare OTUs showed a sequence similarity of less than 95% to a reference sequence.

### DNA vs. cDNA

No pronounced differences between the DNA and cDNA samples of station DY12 could be revealed by the partitioning of diversity analysis (**Supplementary Figure S3**). ANOSIM analyses confirmed this observation, showing no significant disparity between the two sample types (*r* = -0.06; *p* = 0.61). The similarities of paired communities derived from DNA or cDNA sequencing were approximately 90% in the photic layer samples and about 80% in the aphotic layer samples.

Compared with DNA sequencing, cDNA sequencing detected similar numbers of OTUs and relative abundances of different ciliate classes (**Figure 5**). However, Phyllopharyngea and Litostomatea were more diverse and abundant in the cDNA samples than in the DNA samples, with average contributions of 5.4 and 2.9%, respectively, to the total OTUs in each layer (**Figure 5** and **Supplementary Figure S3**).

OTUs that were unique to the dataset of DNA sequencing always occurred in low sequence numbers. Similarly, in the photic layer samples, only the OTUs unique to the cDNA-dataset were in low abundance, whereas, in the aphotic layer samples, some of the unique OTUs appeared to be relatively abundant.

## Discussion

### Methodological Consideration: DNA vs. cDNA

Previous studies indicated that differences in environmental conditions, e.g., temperature and pressure, of coastal and deep-sea sediments might lead to an obvious variation in the similarity between the communities derived from DNA or cDNA sequencing (Massana et al., 2015; Zhao and Xu, 2016). We, for the first time, show that the community structures of pelagic ciliates detected by DNA or cDNA sequencing are similar to each other in the open sea from the surface to the abyssopelagic zones. One major exception is phyllopharyngeans, which were more diverse and abundant in the community derived from cDNA sequencing. This might be largely due to the differences inherent to the DNA/cDNA-based techniques. In addition, the large variation in the rRNA gene copy number across species might also affect the DNA/cDNA comparisons in ciliate diversity (Prokopowich et al., 2003; Gong et al., 2013). The higher proportion of phyllopharyngean cDNA sequences compared with the proportion of DNA sequences might be partially explained by a low rRNA gene copy number of some phyllopharyngean taxa detected in our study.

The slightly higher dissimilarity between the DNA and cDNA samples of the bathypelagic and abyssopelagic zones, as observed in our study, might indeed be a result of the accumulation of inactive ciliate cells in these respective layers. However, the difference of community composition derived from DNA and cDNA sequencing did not overwhelm the natural variation among samples, as the community data was still strongly grouped by sampled layers. Therefore, DNA appears to be a reliable genetic tool to investigate the diversity and distribution patterns of pelagic ciliates in the open ocean.

### Distribution of Pelagic Ciliates in Relation to Contemporary Environmental Parameters and Geographic Distance

Coupling next-generation sequencing with statistical analyses, our study revealed a distinct vertical distribution of pelagic ciliate communities from the surface to the abyssopelagic zone in the Western Pacific Ocean, rather than a separation by geographic location. Similar patterns have already been detected for bacterial communities in the Pacific Ocean (Walsh et al., 2016).

The spatial distribution pattern of marine ciliate communities is strongly linked to a stratification of the water column, indicating that environmental factors might be responsible for the observed partitioning of ciliate diversity. Indeed, temperature and chlorophyll a emerged as particularly strong factors shaping the ciliate communities in the oceans. The influence of temperature on pelagic ciliates was also observed in coastal waters (Grattepanche et al., 2016). Temperature could significantly influence the fertility, survival, and feeding of pelagic ciliates, and thus, could control the densities and community composition of ciliates (Finlay, 1977; Weisse et al., 2002). The significant impact of chlorophyll a is quite reasonable, as it generally represents the biomass of pico- and nano-phytoplankton, which are an important part of the food supply for predatory ciliates (Dolan and Marrasé, 1995; Gimmler et al., 2016).

In contrast, geographic distance seems to have little to no effect on the dispersal of marine pelagic ciliates over a spatial scale of 1,300 km, which is consistent with the finding of previous studies in the open sea (Dolan et al., 2012; Gimmler et al., 2016). This is clearly different from the distribution pattern of protist communities in sediments or soils, which are known to diverge considerably across continents (Bates et al., 2013; Fiore-Donno et al., 2016) and also across coastal sediments at the scales of 50 m, 200 km, and 1,000 km (Zhao and Xu, 2017). The proportion of common ciliate species in the surface water layer is high (26.7% of the total OTUs retrieved from all nine surface samples), which might explain why geographic distance has no effect on the dispersal of pelagic ciliates. However, our findings do not imply that pelagic ciliates tend to have a cosmopolitan distribution, as proposed by Finlay (2002), as about 30% of ciliates OTUs were only detected at one layer, or in less than three of the 23 samples. On the other hand, only 12% of the total OTUs could be detected at all sampled layers.

### Vertical Distribution of Pelagic Ciliate Communities

Photic zone: The highest ciliate diversity was encountered in the DCM layer of the Western Pacific Ocean. This is consistent with the investigation of ciliate communities in the European coastal waters (Forster et al., 2016). Ciliates predominantly feed on pico- and nano-autotrophic taxa, and might consume up to 50% of the chlorophyll a biomass in ocean waters (Dolan and Marrasé, 1995). Thus, water layers with a high chlorophyll a content could possibly attract a high diversity of predatory ciliates (Dolan and Marrasé, 1995; Gimmler et al., 2016). This is in agreement with the finding of a larger proportion of nassophorean OTUs/sequences in this layer than in other layers, most of which are predominantly characterized by an algivorous feeding type (Lynn, 2008). Moreover, species richness in the photic zone samples was expected to be higher than those from deep water, because more favorable conditions than those found in the deep layers support a wide range of niches, which can be filled by organisms differing in their functional capacities, and would allow diversification (Monier et al., 2015).

The 200 m transition layer: The 200 m water layer, which represents a depth of major transitions of temperature, dissolved oxygen, salinity, and major nutrients, was also inhabited by a high diversity of pelagic ciliates. Dynamic environmental conditions might result in rapid speciation (Ghiglione et al., 2012; Pontarp et al., 2013). Meanwhile, the sharp chemical/physical gradients might prevent a strong dominance by any particular species, and support a wide range of niches (Schnetzer et al., 2011). This is in agreement with the finding that a low average proportion of abundant taxa was detected in the 200 m water layer. Interestingly, the ciliate community composition in the 200 m water layer was more similar to those in the deeper layers, although the 200 m layer was more similar to the photic zone in terms of environmental parameters than to the other aphotic layers. Light might be an important driver of the ciliate community structure. Little light in the 200 m layer might have resulted in a high similarity of its community with other aphotic layers. The high diversity and high proportion of unique OTUs in the 200 m layer might provide potential species, which could live and dominate in bathypelagic and abyssopelagic zones. The result of the heatmap analysis confirmed this hypothesis: the abundant OTUs in the photic layers, in particular some parasitic species, also had a relatively high sequence proportion in the 200 m layer samples, whereas only a few were detected in the surface layer. Moreover, based on the conclusions of Dolan et al. (2007) and Doherty et al. (2010), the observed distribution of planktonic ciliates most closely matches the neutral theory of random colonization from a large species pool. Therefore, we argue that the 200 m layer might be an important “species bank” for bathypelagic and abyssopelagic zones.

Aphotic zone: In contrast to the upper water layers, spatially and temporally constant environmental conditions over longer timescale below 1,000 m support a narrower range of traits (Ghiglione et al., 2012; Monier et al., 2015). Moreover, the presence of less labile organic matter in the deep layers might have precluded the growth of some protist species in these depths (Schnetzer et al., 2011). The relatively low diversity in the deep layers was expected. However, parasitic ciliates were more diverse and abundant in the aphotic zone than in the photic layers in our study, indicating a greater role of parasitic forms in the dark than in the photic zone. The relatively high proportion of heterotrophic protists with parasitic life strategies might be caused by the limited organic carbon export (Edgcomb, 2016; Zoccarato et al., 2016). The extent to which ciliates might participate in different host-specific parasite relationships or might live a different free-living life style at increased depth is unknown. Nonetheless, molecular signatures of parasitic protists contribute significant fractions of many high-throughput sequencing data, suggesting that parasitic protist taxa are taxonomically diverse and likely play an important ecological role in deep ocean habitats. Surprisingly, Colpodea ciliates such as *Aristerostoma* sp. were quite abundant in the deep layer of 2,000 m. Most colpodeans inhabit soil and freshwater environments, and only few species are described from marine habitats, such as *Aristerostoma* sp. (Foissner, 1993). Nonetheless, this is the first report of *Aristerostoma* sp. in deep ocean waters, where it was predominantly abundant, as detected by both DNA and cDNA sequencing. In the future, it should be worthwhile to study the taxonomy, phylogeny, and true abundance of *Aristerostoma* sp. in deep sea by combining fluorescent *in situ* hybridization (FISH), light microscopy, and scanning electron microscopy (SEM), e.g., as done by Orsi et al. (2012).

### High Novelty of Ciliate Genetic Diversity

The application of high-throughput DNA sequencing contributed substantially to the detection of a broader protist diversity from various environments (Stoeck et al., 2010; Pawlowski et al., 2011). In this study, we uncovered a high degree of genetic novelty within the pelagic ciliates of the Western Pacific Ocean, even though open ocean water was thought to represent a relatively low-diversity habitat (Gimmler et al., 2016). We further showed that potential novel taxa were almost equally distributed along a depth gradient, even in the abyssopelagic zone, where the ciliate diversity is far less known than in the photic zone. To discover novel taxa in open ocean water in the future, we should pay more attention to taxa of the photic zone rather than those of the bathypelagic or abyssopelagic zones, as taxa of the photic zone can be collected and cultivated with much more ease than those of the deeper water layers, but contain an equal degree of novel diversity.

In addition, the fact that the most abundant nassophorean OTUs at photic layers had a sequence similarity of less than 90% to reference sequences reflects an obvious gap between the obtained sequences and the reference databases. In the future, more efforts in the isolation, cultivation, and description of protists are necessary to link the environmental sequences to the real protist inventory (Filker et al., 2016; Forster et al., 2016). The design of novel species-specific primers and probes based on the retrieved sequences would also help in the identification of target species through molecular techniques (Orsi et al., 2012; Gimmler and Stoeck, 2015).

## Author Contributions

FZ was the main contributor to the experimental work, analysis of the sequence data and drafted the manuscript. KX contributed to the design of the study. PH and SZ participated in the experimental work. SF participated in the analysis of sequencing data and interpretation of the data. SF, KX, PH, and SZ performed a critical revision of the manuscript. All authors approved the final version of the article.

## Conflict of Interest Statement

The authors declare that the research was conducted in the absence of any commercial or financial relationships that could be construed as a potential conflict of interest.

## Supplementary Material

**FIGURE S1 |** Rarefaction curves for all samples under study.

**FIGURE S2 |** Number of shared and unique OTUs between the pairwise layers as well as five layers of the three stations of DY1, DY7 and DY12.

**FIGURE S3 |** UPGMA clustering based on the Sorensen-Dice similarity coefficient and taxonomic composition of sequences of major ciliate groups.

**TABLE S1 |** The concentrations of total phosphorus (TP), total nitrogen (TN), NO_3_-N, NO_2_-N, PO_4_-P, SiO_3_-Si, and NH_4_-N of water samples collected from the Western Pacific Ocean.

**TABLE S2 |** Number of high-quality ciliate sequences and obtained OTUs, the Shannon index and the effective species number of the different samples.
