# Supplementary figures and images for: Patterns and Drivers of Vertical Distribution of the Ciliate Community from the Surface to the Abyssopelagic Zone in the Western Pacific Ocean
Source: Front Microbiol. 2017 Dec 19;8:2559. doi: 10.3389/fmicb.2017.02559 (PMC5742212; doi:10.3389/fmicb.2017.02559)

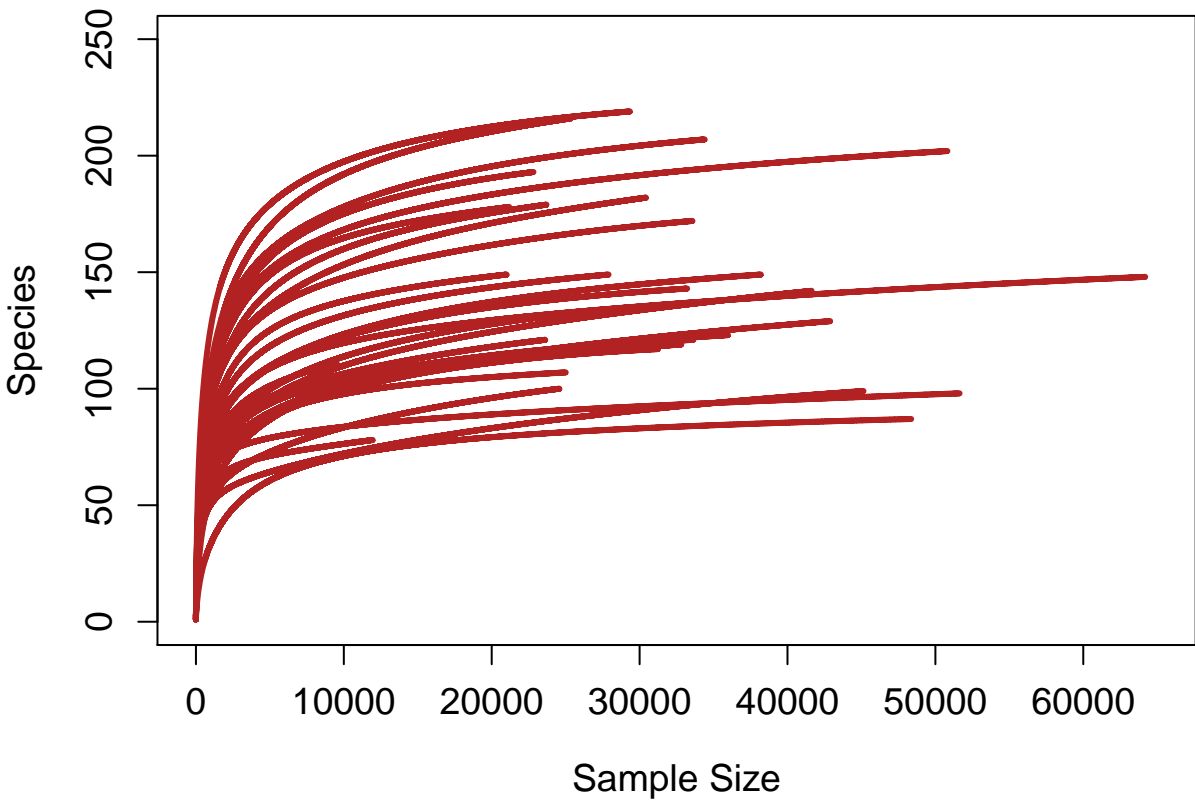

Supplement: Supplementary file 1 [file Image_1.PDF]

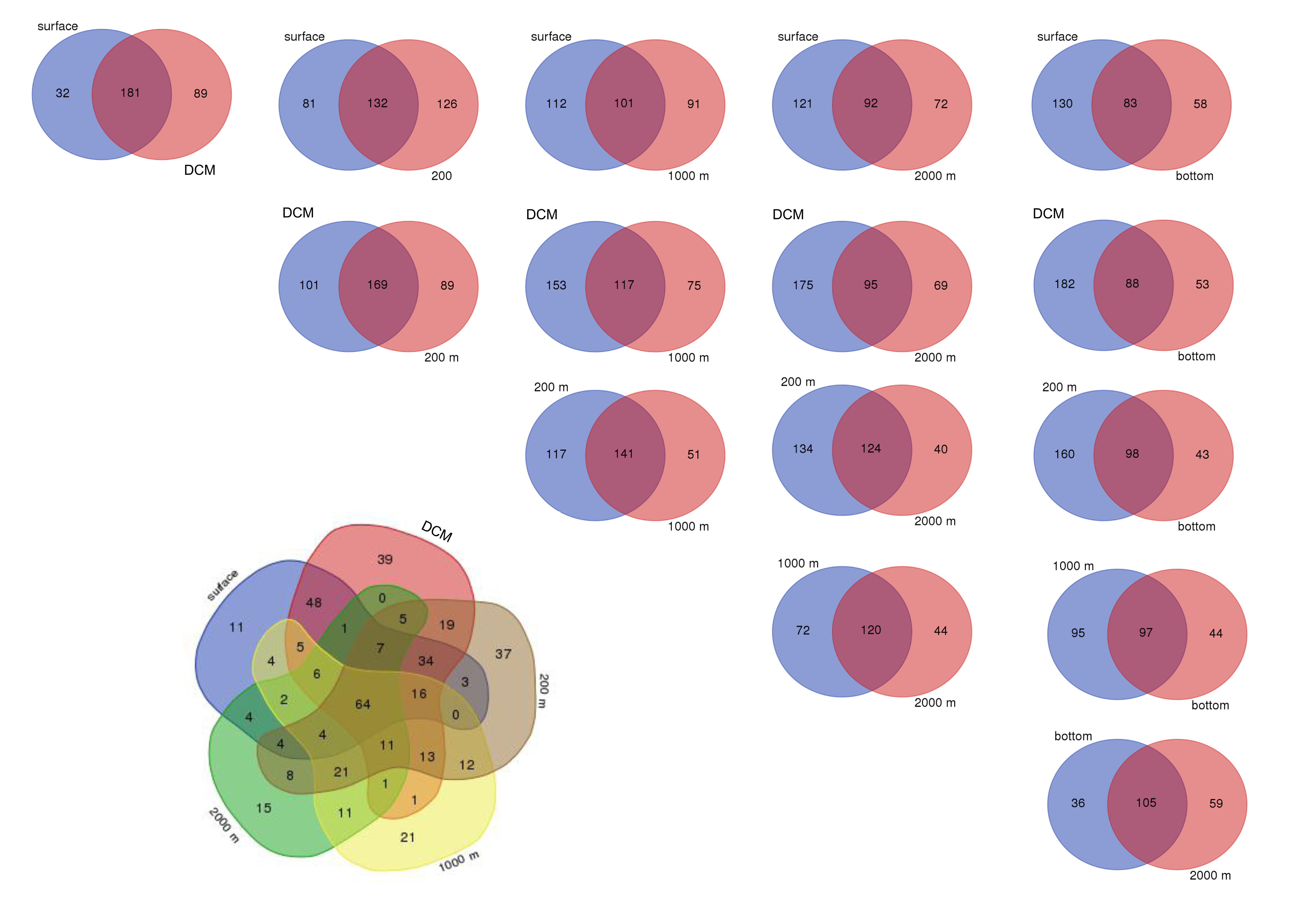

Supplement: Supplementary file 2 [file Image_2.TIF]

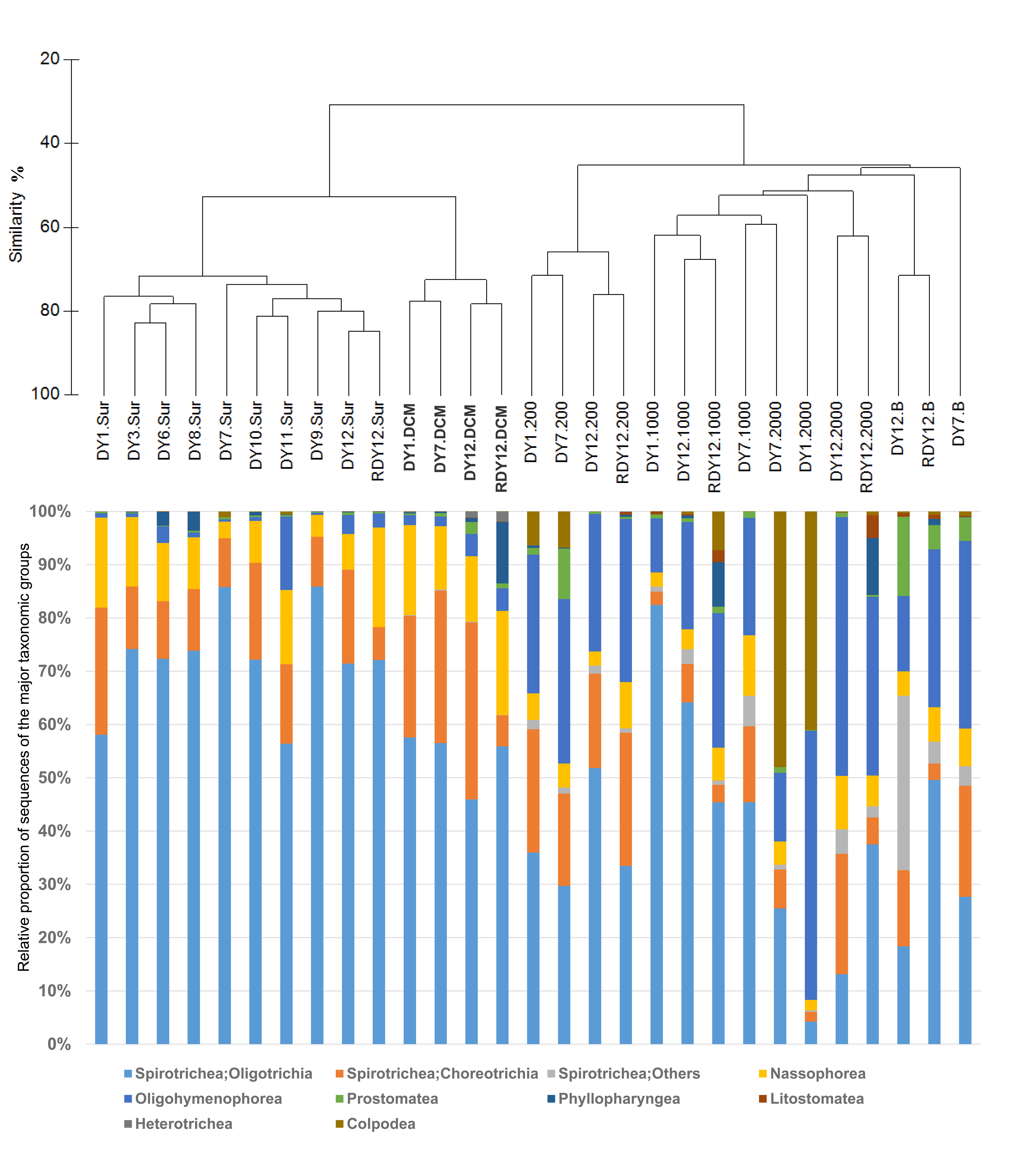

Supplement: Supplementary file 3 [file Image_3.TIF]
