# Supplementary material for: Patterns and Drivers of Vertical Distribution of the Ciliate Community from the Surface to the Abyssopelagic Zone in the Western Pacific Ocean
Source: Front Microbiol. 2017 Dec 19;8:2559. doi: 10.3389/fmicb.2017.02559 (PMC5742212; doi:10.3389/fmicb.2017.02559)
Supplement: Supplementary file 4 [file Table_1.DOCX]

**Table S1** The concentrations of total phosphorus (TP), total nitrogen (TN), NO_3_-N, NO_2_-N, PO_4_-P, SiO_3_-Si, and NH_4_-N of water samples collected from the Western Pacific Ocean.

| Samples | TP (µg/L) | TN (µg/L) | PO_4_-P (µg/L) | NO_3_-N (µg/L) | NO_2_-N (µg/L) | SiO_3_-Si (µg/L) | NH_4_-N (µg/L) |
| --- | --- | --- | --- | --- | --- | --- | --- |
| DY3.Sur | 5.88 | 113 | 0.84 | 1.17 | 0.79 | 29.4 | 26.1 |
| DY6.Sur | 5.96 | 135 | 2.86 | 6.21 | 0.73 | 35.8 | 31.2 |
| DY8.Sur | 7.34 | 137 | 2.00 | 1.68 | 0.40 | 47.1 | 18.9 |
| DY9.Sur | 3.42 | 67.4 | 0.83 | 5.79 | 0.14 | 21.4 | 12.8 |
| DY10.Sur | 7.34 | 133 | 1.92 | 5.47 | 0.90 | 58.6 | 9.48 |
| DY11.Sur | 86.5 | 626 | 2.71 | 20.3 | 0.94 | 50.8 | 8.39 |
| DY1.Sur | 8.05 | 159 | 2.75 | 1.87 | 0.35 | 34.4 | 18.2 |
| DY1.DCM | 6.05 | 113 | 1.49 | 1.79 | 1.06 | 62.5 | 9.94 |
| DY1.200 | 9.77 | 164 | 6.61 | 34.5 | 0.95 | 99.6 | 7.11 |
| DY1.1000 | 66.4 | 506 | 78.76 | 516 | 1.48 | 4158 | 18.7 |
| DY1.2000 | 90.3 | 709 | 80.48 | 609 | 1.26 | 3316 | 18.6 |
| DY7.Sur | 8.06 | 156 | 78.33 | 538 | 1.30 | 1993 | 21.3 |
| DY7.DCM | 8.59 | 112 | 2.95 | 2.16 | 1.03 | 55.6 | 6.21 |
| DY7.200 | 21.6 | 327 | 4.58 | 1.99 | 1.21 | 61.9 | 11.9 |
| DY7.1000 | 103 | 625 | 82.48 | 565 | 1.39 | 2684 | 17.1 |
| DY7.2000 | 93.3 | 624 | 83.53 | 580 | 1.69 | 2963 | 25.2 |
| DY7.B | 40.9 | 294 | 76.50 | 550 | 1.55 | 2869 | 24.9 |
| DY12.Sur | 7.99 | 251 | 1.65 | 15.2 | 0.47 | 40.4 | 9.07 |
| DY12.DCM | 6.49 | 120 | 2.50 | 10.8 | 1.13 | 47.6 | 8.71 |
| DY12.200 | 90.2 | 667 | 39.14 | 199 | 1.76 | 442 | 0.07 |
| DY12.1000 | 87.5 | 719 | 83.72 | 582 | 0.96 | 2436 | 14.0 |
| DY12.2000 | 90.5 | 815 | 83.11 | 587 | 1.08 | 2922 | 14.7 |
| DY12.B | 80.2 | 621 | 74.17 | 541 | 1.23 | 2710 | 11.8 |
