# Supplementary material for: Patterns and Drivers of Vertical Distribution of the Ciliate Community from the Surface to the Abyssopelagic Zone in the Western Pacific Ocean
Source: Front Microbiol. 2017 Dec 19;8:2559. doi: 10.3389/fmicb.2017.02559 (PMC5742212; doi:10.3389/fmicb.2017.02559)
Supplement: Supplementary file 5 [file Table_2.DOCX]

**Table S2** Number of high-quality ciliate sequences and obtained OTUs, the Shannon index and the effective species number of the different samples.

|  | No. of ciliate sequences | No. of ciliate OTUs (97%) | Shannon index | Effective species number | |
| --- | --- | --- | --- | --- | --- |
| DY3.Sur | 27900 | 149 | 3.14 | 23.2 |  |
| DY6.Sur | 21008 | 149 | 3.14 | 23.1 |  |
| DY8.Sur | 64187 | 148 | 3.28 | 26.7 |  |
| DY9.Sur | 25004 | 107 | 2.86 | 17.5 |  |
| DY10.Sur | 33216 | 143 | 3.09 | 22.0 |  |
| DY11.Sur | 35979 | 123 | 3.20 | 24.6 |  |
| DY1.Sur | 30430 | 182 | 3.57 | 35.6 |  |
| DY1.DCM | 34395 | 207 | 3.53 | 34.1 |  |
| DY1.200 | 21182 | 178 | 3.48 | 32.5 |  |
| DY1.1000 | 33630 | 121 | 1.44 | 4.22 |  |
| DY1.2000 | 48363 | 87 | 1.27 | 3.56 |  |
| DY7.Sur | 24578 | 100 | 2.63 | 13.9 |  |
| DY7.DCM | 50812 | 202 | 3.77 | 43.3 |  |
| DY7.200 | 33575 | 172 | 3.61 | 37.1 |  |
| DY7.1000 | 23619 | 121 | 3.26 | 26.0 |  |
| DY7.2000 | 32817 | 119 | 2.26 | 9.57 |  |
| DY7.B | 51637 | 98 | 3.07 | 21.5 |  |
| DY12.Sur | 27925 | 134 | 3.38 | 29.4 |  |
| DY12.DCM | 29343 | 219 | 4.12 | 61.6 |  |
| DY12.200 | 23704 | 179 | 3.15 | 23.3 |  |
| DY12.1000 | 9513 | 111 | 2.69 | 14.7 |  |
| DY12.2000 | 11954 | 78 | 3.12 | 22.7 |  |
| DY12.B | 45138 | 99 | 2.64 | 14.1 |  |
| RDY12.Sur | 31236 | 117 | 2.86 | 17.5 |  |
| RDY12.DCM | 22823 | 193 | 3.71 | 40.8 |  |
| RDY12.200 | 25338 | 216 | 3.82 | 45.5 |  |
| RDY12.1000 | 38202 | 149 | 3.26 | 26.0 |  |
| RDY12.2000 | 41632 | 142 | 3.18 | 24.1 |  |
| RDY12.B | 42885 | 129 | 2.85 | 17.2 |  |
